# Hypoxic microenvironment-induced exosomes confer temozolomide resistance in glioma through transfer of pyruvate kinase M2

**DOI:** 10.1007/s12672-024-00963-9

**Published:** 2024-04-10

**Authors:** Guofu Li, Ziyu Xiong, Ying Li, Cong Yan, Yingying Cheng, Yuwen Wang, Jingwei Li, Zifeng Dai, Dongdong Zhang, Wenzhong Du, Chunyang Men, Changbin Shi

**Affiliations:** 1 Department of Neurosurgery, Harbin Medical University Cancer Hospital, Harbin, Heilongjiang China; 2 Department of Neurosurgery, The First Affiliated Hospital of Harbin Medical University, Harbin, Heilongjiang China; 3 Department of Ultrasound, Harbin Medical University Cancer Hospital, Harbin, Heilongjiang China

**Keywords:** Glioma, Hypoxia, Exosome, Pyruvate kinase M2, Temozolomide, Resistance

## Abstract

**Objective:**

Glioma, a malignant primary brain tumor, is notorious for its high incidence rate. However, the clinical application of temozolomide (TMZ) as a treatment option for glioma is often limited due to resistance, which has been linked to hypoxic glioma cell-released exosomes. In light of this, the present study aimed to investigate the role of exosomal pyruvate kinase M2 (PKM2) in glioma cells that exhibit resistance to TMZ.

**Methods:**

Sensitive and TMZ-resistant glioma cells were subjected to either a normoxic or hypoxic environment, and the growth patterns and enzymatic activity of glycolysis enzymes were subsequently measured. From these cells, exosomal PKM2 was isolated and the subsequent effect on TMZ resistance was examined and characterized, with a particular focus on understanding the relevant mechanisms. Furthermore, the intercellular communication between hypoxic resistant cells and tumor-associated macrophages (TAMs) via exosomal PKM2 was also assessed.

**Results:**

The adverse impact of hypoxic microenvironments on TMZ resistance in glioma cells was identified and characterized. Among the three glycolysis enzymes that were examined, PKM2 was found to be a critical mediator in hypoxia-triggered TMZ resistance. Upregulation of PKM2 was found to exacerbate the hypoxia-mediated TMZ resistance. Exosomal PKM2 were identified and isolated from hypoxic TMZ-resistant glioma cells, and were found to be responsible for transmitting TMZ resistance to sensitive glioma cells. The exosomal PKM2 also contributed towards mitigating TMZ-induced apoptosis in sensitive glioma cells, while also causing intracellular ROS accumulation. Additionally, hypoxic resistant cells also released exosomal PKM2, which facilitated TMZ resistance in tumor-associated macrophages.

**Conclusion:**

In the hypoxic microenvironment, glioma cells become resistant to TMZ due to the delivery of PKM2 by exosomes. Targeted modulation of exosomal PKM2 may be a promising strategy for overcoming TMZ resistance in glioma.

**Supplementary Information:**

The online version contains supplementary material available at 10.1007/s12672-024-00963-9.

## Introduction

Glioma is the most prevalent primary malignant tumor in the brain, accounting for approximately 78% of all malignant cases. It arises from glial cells [1]. Based on the 2021 WHO classification of tumors of the central nervous system, gliomas are classified as different subtypes, including diffuse astrocytoma, polymorphous low-grade neuroepithelial tumor of the young, diffuse low-grade glioma, diffuse hemispheric glioma, diffuse pediatric-type high-grade glioma, and high-grade astrocytoma [2]. The incidence rate of glioma varies from 1.9 to 9.6 per 100,000 individuals among adults, which is influenced by age, gender, ethnicity, and geographical location [3]. The development of glioma is primarily associated with several risk factors, including advanced age, a family history of the condition, and exposure to radiation. Standard treatment for glioma typically involves a safe surgical resection, followed by the administration of radiotherapy and TMZ chemotherapy [4]. TMZ is an oral alkylating drug with easy penetration through the blood-brain barrier, commonly used as a first-line treatment for glioma [5–7]. However, around 50% of glioma patients experience resistance to TMZ and suffer relapse after a progression-free survival period of 7–10 months [8]. Moreover, many patients encounter severe side effects, such as bone marrow inhibition and female infertility [8]. However, the potential mechanisms involved in TMZ resistance remained largely unclear. Therefore, further elucidation of the mechanisms underlying TMZ resistance in glioma is imperative.

Gliomas are characterized by pseudopalisading necrosis, a pathological feature commonly seen near collapsed blood vessels, surrounded by cells that survived in hypoxic regions [9]. Hypoxia promotes the Warburg effect, an abnormal glycolytic process resulting in increased glucose uptake and lactate production [10]. This process provides biosynthesis precursors of nucleotides, amino acids, and lipids essential for supporting rapid tumor growth [11]. Pyruvate kinase (PK) is a critical enzyme in glycolysis, catalyzing the final rate-limiting step by transferring phosphate to generate pyruvate and adenosine triphosphate from phosphoenolpyruvate and adenosine diphosphate [12]. Among the four human PK isoforms (M1, M2, L, and R), PKM2 is abundantly expressed in glioma cells but is marginally expressed in healthy brain tissues [13]. TMZ-induced DNA damage is linked to PKM2 because of its influence on pyruvate metabolism [14], though the role of PKM2 in TMZ resistance remains obscure. Exosomes, small membrane vesicles, are secreted by specific cell types. Constituting a vital element of the tumor microenvironment, exosomes encapsulate diverse biomolecules, including mRNAs, non-coding RNAs, and proteins. They play a pivotal role in intercellular communication by transmitting their contents [15]. Research has shown that hypoxia modifies the release and contents of exosomes, impacting intercellular communication in glioma [16]. In particular, exosomal circ_0072083 induced by the Warburg effect enhances TMZ resistance in glioma [16], and hypoxic glioma-released exosomes containing microRNA-1246 induce M2 macrophage polarization [17]. Exosomes delivered by hypoxic glioma stem cells containing Linc01060 promote glioma progression [18]. All of these research indicates that exosomes have an significant relationship with gliomas, and it is very possible that exosomes are involved in the process of resistance to TMZ in gliomas. While there is limited evidence suggesting the release of PKM2-containing exosomes by tumor cells, its specific role in TMZ-resistant glioma remains unclear. This study predominantly centers on exploring exosomes transported by the hypoxic microenvironment, elucidating their role in conferring TMZ resistance to glioma cells through the delivery of PKM2. This investigation aims to unveil a novel mechanism of TMZ resistance in glioma, proposing a potential metabolic blocker for effective anti-glioma therapy.

## Materials and methods

### Cell culture and administration

U251 cells (Procell, Wuhan, China) were used to contain 10% fetal bovine serum (FBS; HyClone, USA) and 1% penicillin/streptomycin in Dulbecco’s modified eagle medium (DMEM) at 37 °C. U251/TR resistant cell lines are obtained by exposing U251 cells to increasing concentrations of TMZ until cells are resistant to TMZ at a concentration of 50 μg/mL [16]. These cells are typically cultured in a normoxic (21% O_2_) or hypoxic (1% O_2_) environment.

### Tumor-associated macrophages (TAMs) induction

U937cells (Procell, Wuhan, China) are inoculated into 10-cm culture dishes (5 × 10 ^5^ cells/well) with RPMI-1640 (10% FBS, 100 ng/mL of PMA). After 24 h of culture, cells were washed with phosphate-buffered saline (PBS, pH 7.2) and re-culture with 10%-FBS RPMI, 20 ng/ml IL-4 (Gibco, Thermo) were added for co-incubation. Then cells were washed with PBS and incubated with serum-free 1640 for 24 h. After 24 h serum starvation, cells were collected for M2-polarized TAMs use. Cell morphology was observing for successful induction demonstration which the cell morphology was changed from round shape to long spindle shape. Some of the cells having “tentacles” protruding out, consistent with differentiation into macrophages. Exosomes at a concentration of 1 μg/mL were added to the medium of recipient cells according to the established protocol.

### Cell viability assay

Cell viability experiments were performed using the Cell Counting Kit-8 (CCK-8; Solarbio, China). For each well of a 96-well plate, a uniform seeding of 2 × 10^3^ cells was carried out. Following cell adhesion, 10 μL of CCK-8 reagent was added to each well. The cells were then incubated in a standard cell culture incubator for 2 h, allowing the CCK-8 reagent to interact with cellular dehydrogenases and formazan crystals to develop. After the incubation period, the absorbance value of the formazan product was measured at 450 nm using a microplate reader. This wavelength was selected as it corresponds to the peak absorbance of the formazan product. These steps were consistently applied across all experimental conditions to ensure the reliability and reproducibility of the cell viability assessments.

### Colony formation assay

The ability of the cells to form colonies was assessed by a colony formation assay. 500 cells were inoculated in each well of a 6-well plate separately and cultured for 10 days. The resulting colonies were then fixed and stained with 0.5% crystal violet (Sigma-Aldrich, USA), then images of colony formation were taken and the number of colonies was quantified.

### Glucose uptake assay

2-Deoxyglucose uptake assay reagent (Sigma-Aldrich, USA) was performed. Specifically, cells were inoculated into six confluent plates at a cell density of 7 × 10^4^ cells per tool. Cells were washed with PBS and incubated for 1 h in a glucose-free incubator, then treated with 1 mm 2-deoxyglucose for 20 min. Glucose uptake was determined by measuring absorbance at 540 nm.

### Lactate production assay

Cells were seeded at a density of 5 × 103 cells per well in 96-well plates to measure lactate production. Human lactate concentration kit (Nanjing Jiancheng Bioengineering Institute,China) was used for calculating lactate concentration.

### RT-qPCR

Trizol (invitrogen, USA) was used to extract a common RNA from cells with subsequent reverse transcription using applied biosystems (USA) reverse transcription set. RT-PCR was performed with the quantitative PCR mx-3000p (Agilent, USA), where primers are specific to the following target genes: PKM2 (Forward 5′-ATGTCGAAGCCCCATAGTGAA-3′, Reverse 5′-TGGGTGGTGAATCAATGTCCA-3′), HK2 (Forward 5′-GAGCCACCACTCACCCTACT-3′, Reverse 5′-CCAGGCATTCGGCAATGTG-3′), LDHA (Forward 5′-ATGGCAACTCTAAAGGATCAGC-3′, Reverse 5′-CCAACCCCAACAACTGTAATCT-3′), and β-actin (Forward 5′-CATGTACGTTGCTATCCAGGC-3′, Reverse 5′-CTCCTTAATGTCACGCACGAT-3′). In our study, we employed the 2-ΔΔCT method for relative quantification of gene expression. This method involves calculating the difference in cycle threshold (CT) values between the target gene and the reference gene, using the control group as a baseline. Subsequently, it calculates the relative expression levels, providing a reliable analytical framework for our experiments that takes into account the influences of experimental errors and amplification efficiency.

### Immunoblotting

Lysis buffer was determined by radioimmunoprecipitation and protein concentration by the bovine serum albumin method. Proteins were isolated from the lysate by SDS-PAGE and transferred to a PVDF membrane. After containment, specific primary resistance to PKM2 (1/1000; ab85555; Abcam, USA), HK2 (1/1000; Ab133691), LDHA (1/5000; Ab52488), CD36 (1/1000; Ab133625), TSG101 (1/1000; Ab133586), PARP (1/1000; Ab32071), Bax (1/1000; Ab32503), bcl-2 (1/2000; Ab182858) or beta-actin (1/200; Ab115777) for incubation followed by peroxidase-coupled secondary resistance for incubation. Final detection of protein traces using an improved chemiluminescence detection system (Pierce, USA).

### Immunofluorescence

After 20 min immobilization with 4% paraformaldehyde (Sigma-Aldrich, USA), cells were permeated with 0.5% Triton x-100 (Sigma-Aldrich, USA) for 10 min. Rinse the solution, then seal the cells with 0.1% BSA prepared by PBS for 30 min. Subsequently, anti-pkm2 antibody (1/100; Ab85555; Abcam, USA) cells are incubated overnight at 4 °C. Next, the cell is coupled to Alexa Fluor^®^ 594 with secondary resistance (1/200; Ab150080; Abcam) are incubated together and finally with DAPI (ab104139; Abcam) dye. Cell fluorescence images were taken with a fluorescence microscope (Leica, Germany).

### Plasmids and virus infection

The PKM2-CopGFP fusion gene (GenePharma, China) was used to achieve overexpression of PKM2 (OE-PKM2). A complete PKM2-CopGFP series was inserted into the Lv5 lentiviral vector. At the same time, we adopted PKM2 targeting (shPKM2; GenePharma, China) to silence PKM2. The sequences targeted were: scramble shRNA, AATGCACGCTCAGCACAAGC; PKM2-shRNA1, GTTCGGAGGTTTGATGAAATC; PKM2-shRNA2, GCTGTGGCTCTAGACACTAAA. After production and purification, lentiviral particles are stored at − 80 °C. fusion gene (GenePharma, China) was used to achieve overexpression of PKM2 (OE-PKM2). A complete PKM2-CopGFP series was inserted into the Lv5 lentiviral vector. At the same time, we adopted PKM2 targeting (shPKM2; GenePharma, China) to silence PKM2. After production and purification, lentiviral particles are stored at − 80 °C.

### Isolation of tumor-derived exosomes

Following cell collection and centrifugation, the medium containing serum was aspirated, and the cells were washed twice with PBS. Subsequently, the cells were incubated with serum-free medium for 48 h. Dead cells and cell debris were then removed by centrifugation at 300 g, 2000 g and 10,000 g at 4 °C for 10 and 30 min. The resulting supernatant was filtered through a 0.22 μm filter and centrifuged at 120,000 g for 70 min at 4 °C. After discarding the supernatant, the pellet was suspended in PBS to obtain exosomes.

### Exosome uptake assay

We use the red fluorescent dye PKH26 (Sigma, USA) for fluorescent labeling of exosomes. Initially, resuspension was performed using 100 μL of exogenous diluent C and mixing was performed by adding 100 μ of LPKH26 dye reagent. After 3 min of incubation, 200 μL of serum was added and washed with PBS. Finally, the exosome was cocultured with the recipient cell and photographed.

### Flow cytometry

To assess apoptotic cells, the Accuri C6 flow cytometer (BD, USA) was employed, following labeling with Annexin V-FITC and propidium iodide (PI) (CA1020; Solarbio, China). Cells were harvested, washed with PBS, and suspended in a binding buffer for Annexin V-FITC and PI staining, as per the manufacturer's instructions. After incubation, the labeled cell suspension underwent flow cytometry analysis using appropriate laser and filter settings to detect Annexin V-FITC and PI fluorescence. Data were analyzed using FlowJo software to quantify apoptotic and necrotic cell populations.

### Intracellular reactive oxygen species (ROS) measurement

ROS levels were monitored using a fluorescent determination of 2', 7'-dichloroflucéine diacetate (dcfh-da), according to the specifications provided by the manufacturer (D6470; Solarbio, China).

### Transwell

Cell invasion experiments were conducted using a Matrigel-coated invasion chamber (BD, USA). Cells were inoculated into the upper chamber of the device, and a medium containing 20% FBS was added to the lower chamber. After 24 h of culture, cells that had penetrated the bottom surface through the filter were fixed and stained with 0.5% crystal violet.

### Statistical analysis

Data are expressed as mean ± standard deviation. Statistical analysis was performed using GraphPad Prism software (version 9.0.1). Half of the maximum inhibitory concentration of TMZ (CI50) was determined and two groups were compared using Student’s t-test, while three or more groups were compared using one-way or two-way ANOVA. Differences in p-values less than 0.05 were considered statistically significant.

## Results

### The hypoxic microenvironment deteriorates TMZ resistance in glioma cells

**Fig. 1:**
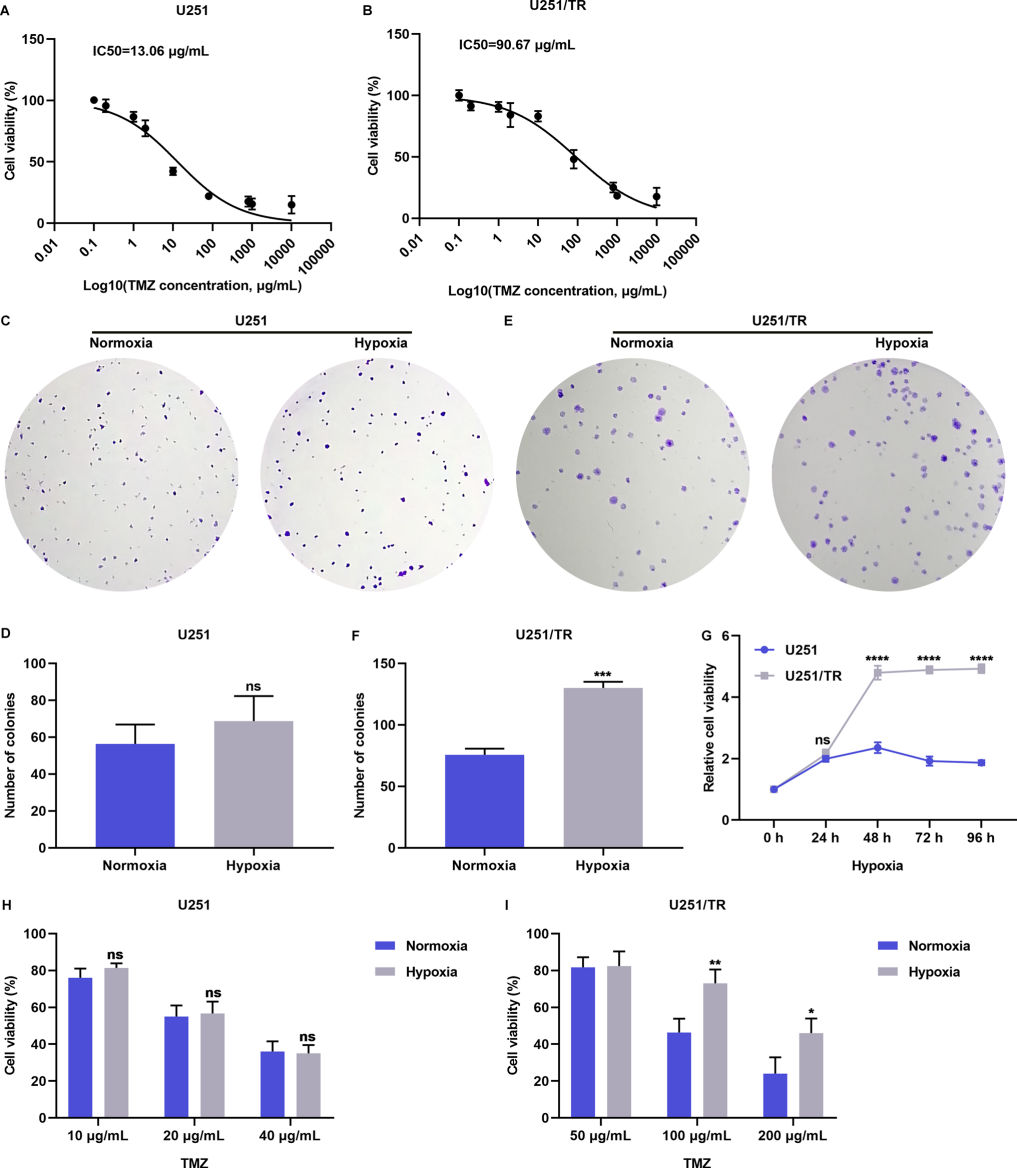
Hypoxia deteriorates TMZ resistance in glioma cells. **A**, **B** IC50 curves of U251 and U251/TR cells exposed to distinct doses of TMZ for 48 h. **C**–**F** Number of colonies of U251 and U251/TR cells with normoxic and hypoxic conditions. **G** Cell viability of U251 and U251/TR cells with hypoxic conditions for distinct time periods. **H**, **I** Cell viability of U251 and U251/TR cells with normoxic and hypoxic conditions and administrated with distinct doses of TMZ for 48 h. Ns: no significance; *p < 0.05; **p < 0.01; ***p < 0.001; and ****p < 0.0001

In this study, we aimed to investigate the impact of hypoxic conditions on TMZ resistance in glioma cells. To accomplish this, we established a TMZ-resistant U251 cell line (U251/TR) and found that it showed higher resistance to TMZ compared to the parental U251 cells, as evidenced by IC50 curves (Fig. 1A, B). We then investigated the effect of normoxic and hypoxic conditions on the proliferative capacity of U251 cells but did not observe any significant differences between the two conditions (Fig. 1C, D). Interestingly, however, colony formation assays revealed a significant increase in the number of colonies formed by U251/TR cells under hypoxic conditions compared to those under normoxic conditions (Fig. 1E, F), implying an association between hypoxia and TMZ resistance in glioma cells. Furthermore, we observed that U251/TR cells had enhanced hypoxic tolerance compared with U251 cells, suggesting that hypoxia is common in TMZ-resistant glioma tumors (Fig. 1G). Notably, when U251 and U251/TR cells were treated with differing doses of TMZ under normoxic or hypoxic conditions for 48 h, the hypoxic microenvironment exacerbated the resistance of U251/TR cells to TMZ but did not have any effect on the response of U251 cells to the drug (Fig. 1H, I). Taken together, our results suggest that the hypoxic microenvironment contributes to the development of TMZ resistance in glioma cells.

### PKM2 is a crucial mediator in hypoxia-triggered TMZ resistance of glioma cells

**Fig. 2:**
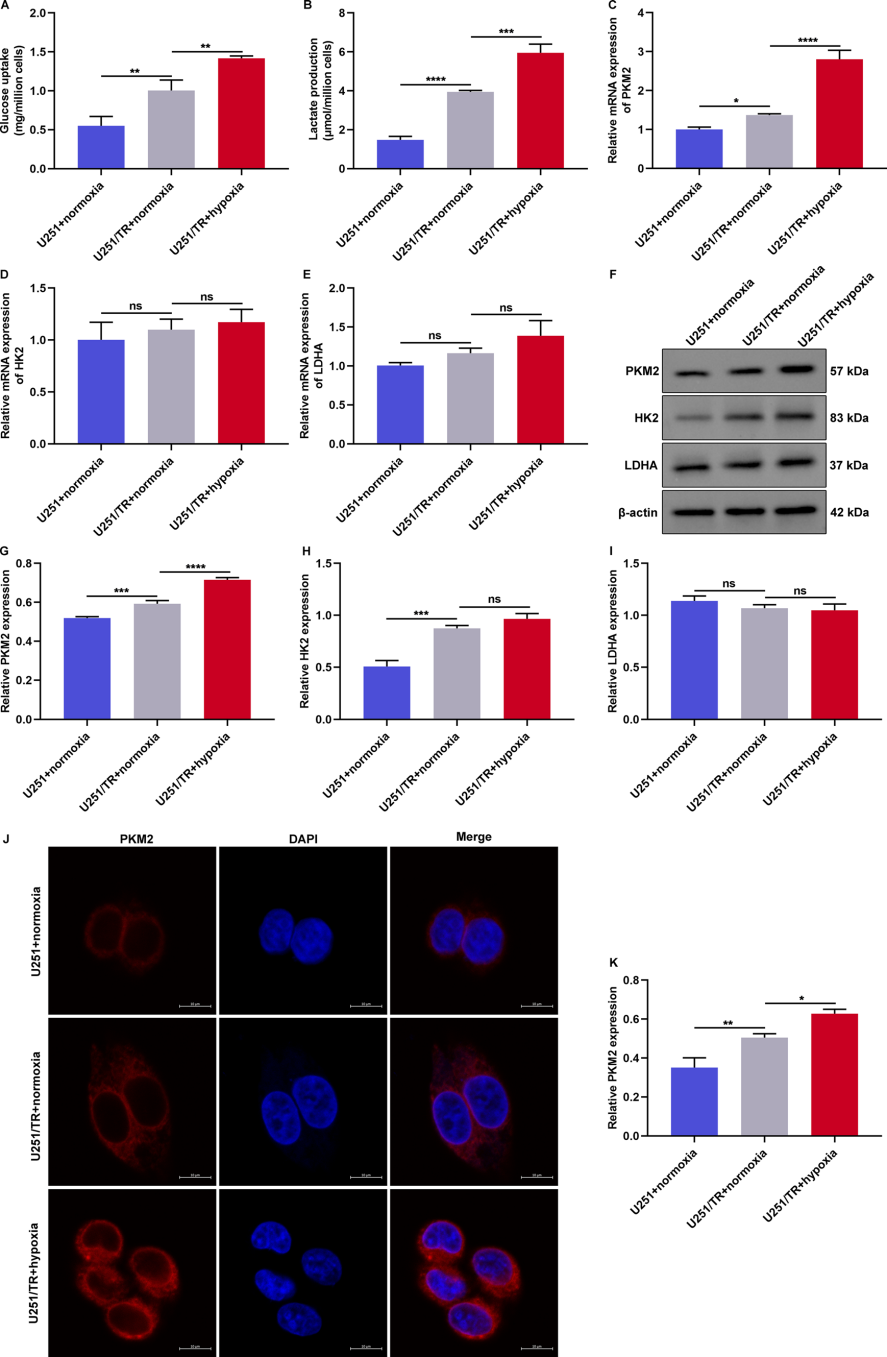
PKM2 is a crucial mediator in hypoxia-triggered TMZ resistance of glioma cells. **A**, **B** Glucose uptake and lactate production of U251 cells with normoxia exposure for 48 h, and U251/TR cells with normoxia or hypoxia exposure for 48 h. **C**–**E** RT-qPCR of PKM2, HK2, and LDHA mRNAs in above cells. **F**–**I** Immunoblotting of PKM2, HK2, and LDHA proteins in above cells. **J**, **K** Immunofluorescence of PKM2 in above cells. Bar, 10 μm. Ns: no significance; *p < 0.05; **p < 0.01; ***p < 0.001; and ****p < 0.0001

There is evidence suggesting that the Warburg effect, which is characterized by increased glucose uptake and lactate production, contributes to TMZ resistance in glioma cells [16]. To delve deeper into this phenomenon, we assessed the Warburg effect in U251 cells exposed to normoxia for 48 h and U251/TR cells subjected to either normoxic or hypoxic conditions for the same duration. Our results revealed that U251/TR cells exhibited significantly higher glucose uptake and lactate production compared to U251 cells under normoxic conditions (Fig. 2A, B). Furthermore, exposure to hypoxia intensified these metabolic changes in U251/TR cells. We then measured the expression levels of glycolytic enzymes, including PKM2, HK2, and LDHA. The results showed that PKM2 expression was notably increased in U251/TR cells compared to U251 cells under normoxia (Fig. 2C–I). This upregulation was further exacerbated by exposure to hypoxia. However, no significant changes were observed in the expression levels of HK2 and LDHA in either normoxic U251 cells or normoxic/hypoxic U251/TR cells. Additionally, we found that PKM2 activity was higher in U251/TR cells than in U251 cells, and this difference was further intensified by hypoxia (Fig. 2J, K), indicating that PKM2 may play a critical role in hypoxia-induced TMZ resistance in glioma cells.

### PKM2 exacerbates the hypoxic microenvironment-mediated TMZ resistance of glioma cells

**Fig. 3:**
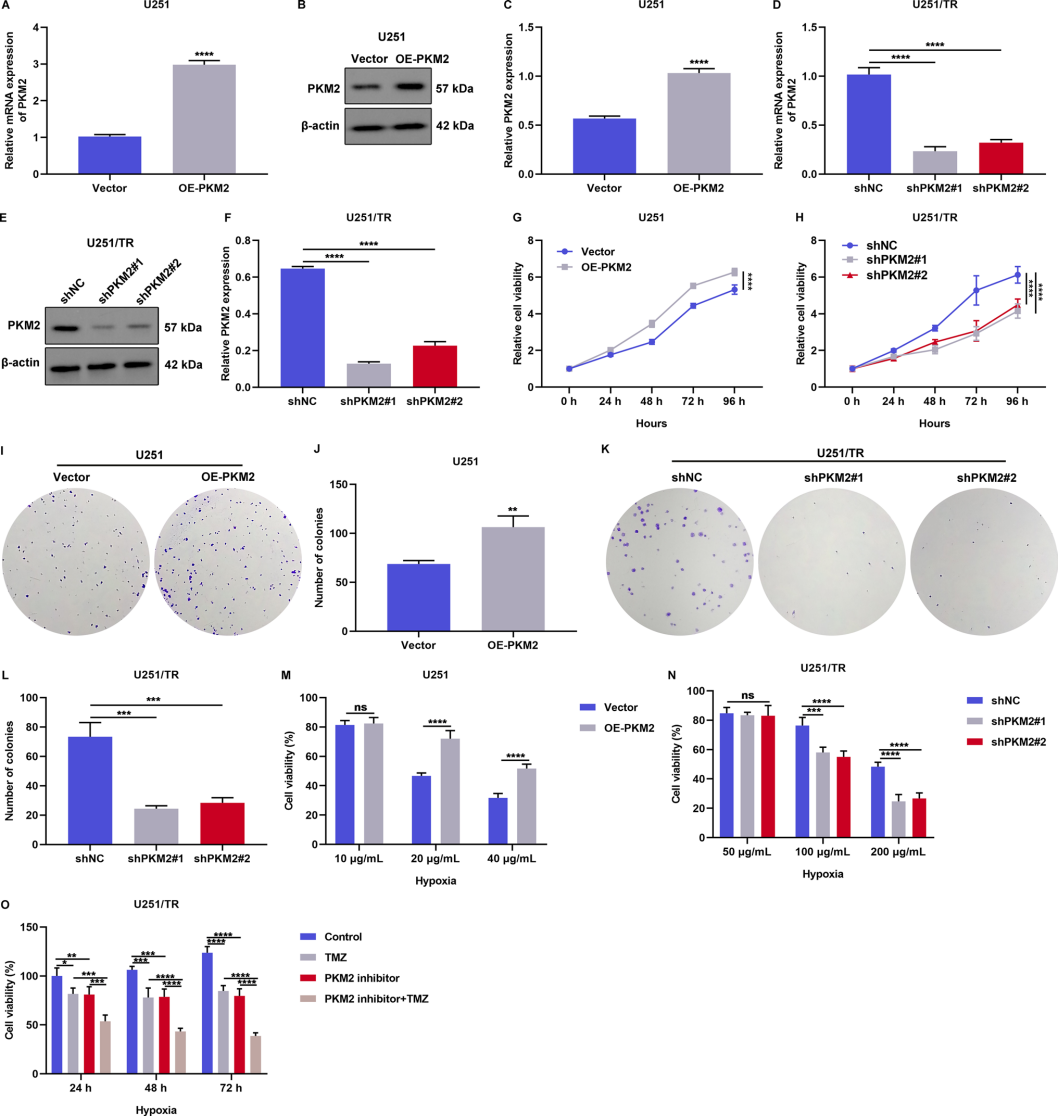
PKM2 exacerbates the hypoxic microenvironment-mediated TMZ resistance of glioma cells. **A**–**C** PKM2 mRNA and protein levels in U251 cells with vector or OE-PKM2 plasmid transfection. **D**–**F** PKM2 mRNA and protein levels in U251/TR cells with shNC or shPKM2 lentivirus transfection. **G**, **H** Cell viability of U251 cells with vector or OE-PKM2 plasmid transfection, and U251/TR cells with shNC or shPKM2 lentivirus transfection. **I**–**L** Number of colonies of U251 cells with vector or OE-PKM2 plasmid transfection, and U251/TR cells with shNC or shPKM2 lentivirus transfection. **M**, **N** Cell viability of vector- or OE-PKM2 plasmid-transfected U251 cells as well as shNC- or shPKM2 lentivirus-transfected U251/TR cells administrated with distinct doses of TMZ in the hypoxic microenvironment. **O** Cell viability of U251/TR cells administrated with 30 μg/mL TMZ, 3 μM PKM2 inhibitor, or their combination for distinct time periods in the hypoxic microenvironment. Ns: no significance; *p < 0.05; **p < 0.01; ***p < 0.001; and ****p < 0.0001

We investigated whether PKM2 plays a role in hypoxia-induced resistance to TMZ in glioma cells. We overexpressed PKM2 in U251 cells by transfecting them with a PKM2 plasmid (Fig. 3A–C), and stably knocked out PKM2 in U251/TR cells using shPKM2 lentivirus transfection (Fig. 3D–F). Our results showed that PKM2 overexpression promoted the growth of U251 cells, whereas its suppression inhibited the survival of U251/TR cells (Fig. 3G–L). In a hypoxic microenvironment, overexpression of PKM2 worsened the resistance of U251 cells to different concentrations of TMZ (Fig. 3M). Conversely, PKM2 knockdown enhanced the response of U251/TR cells to TMZ treatment (Fig. 3N). Furthermore, a combined treatment of TMZ and a PKM2 inhibitor demonstrated a more pronounced therapeutic effect on the survival of U251/TR cells compared to the individual treatments of TMZ or the PKM2 inhibitor alone (Fig. 3O). These findings suggest that PKM2 plays a crucial role in exacerbating hypoxia-induced TMZ resistance in glioma cells.

### Identification of exosomal PKM2 released from hypoxic TMZ-resistant glioma cells

**Fig. 4:**
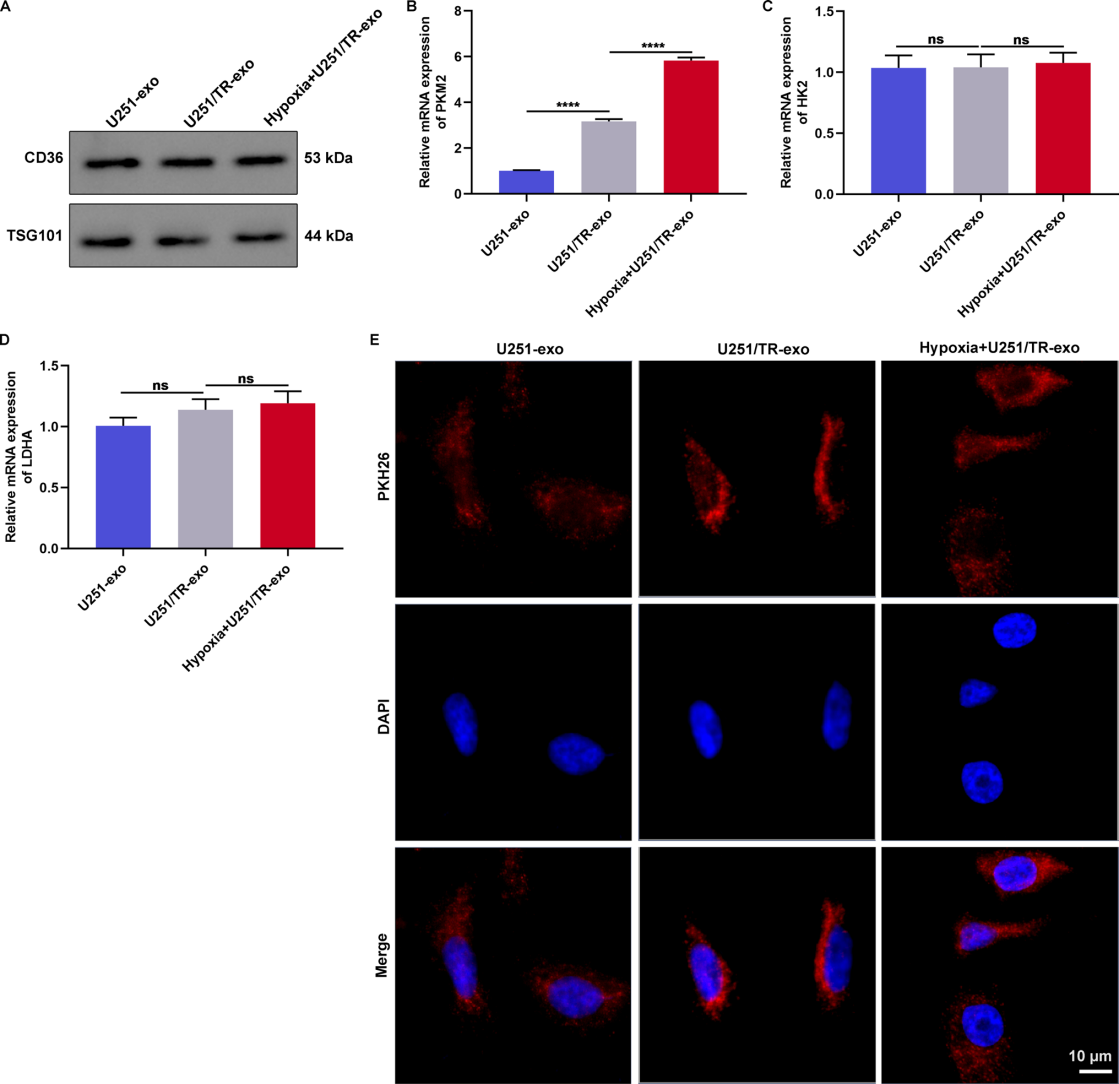
Identification of exosomal PKM2 released from hypoxic TMZ-resistant glioma cells. **A** Immunoblotting of exosomal markers CD63 as well as TSG101 in exosomes isolated from U251 cells, U251/TR cells, and hypoxic U251/TR cells. **B**–**D** RT-qPCR of PKM2, HK2, and LDHA levels in above exosomes. **E** Fluorescence staining of U251 cells with treatment of red fluorescent dye PKH26-labeled exosomes from U251 cells, U251/TR cells, and hypoxic U251/TR cells for evaluating exosome uptake. Bar, 10 μm. Ns: no significance; and ****p < 0.0001

We investigated the role of PKM2 in cell-to-cell communication and transmission of TMZ-resistance through exosomes in glioma. Exosomes were isolated from U251 cells, U251/TR cells, and hypoxic U251/TR cells using ultracentrifugation, which were confirmed to be enriched by exosomal markers CD63 and TSG101 (Fig. 4A), indicating successful isolation of exosomes (Additional file 1: Figure S1). Among the three glycolysis enzymes—PKM2, HK2, and LDHA—PKM2 exhibited higher expression in exosomes released from U251/TR cells than in those released from U251 cells, which was further increased under hypoxia (Fig. 4B). In contrast, no significant differences in HK2 and LDHA levels were observed among exosomes from U251 cells, U251/TR cells, and hypoxic U251/TR cells (Fig. 4C, D). These data suggest that hypoxic TMZ-resistant glioma cells release exosomal PKM2. Additionally, we verified that fluorescent-labeled PKH26 exosomes were effectively internalized by U251 cells (Fig. 4E), indicating that TMZ-sensitive glioma cells can effectively acquire exosomes released from TMZ-sensitive, resistant, and hypoxic glioma cells.

### Exosomal PKM2 released from hypoxic resistant glioma cells transmits TMZ resistance to sensitive glioma cells

**Fig. 5:**
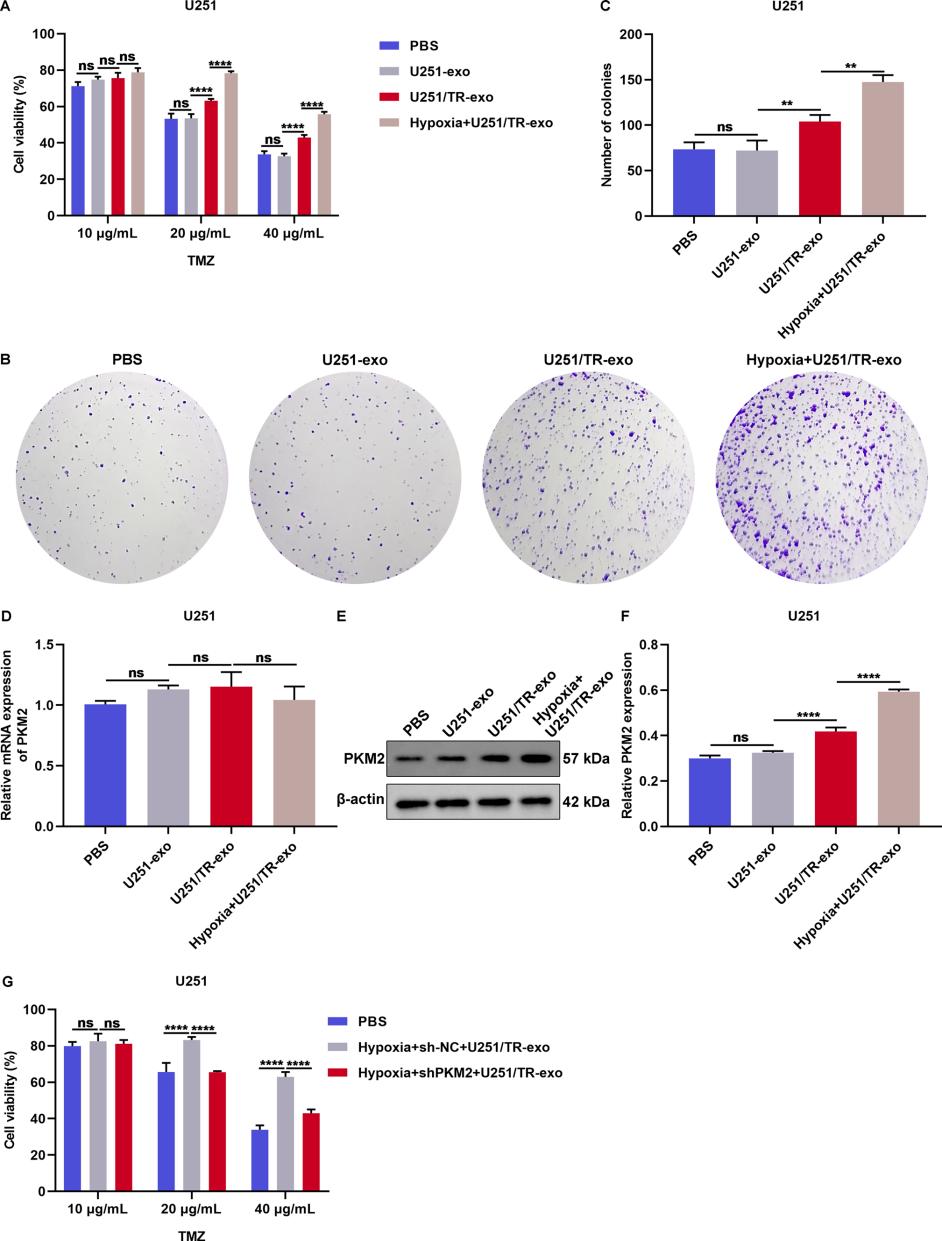
Exosomal PKM2 released from hypoxic resistant glioma cells transmits TMZ resistance to sensitive glioma cells. **A** Viability of U251 cells administrated with PBS or exosomes released from U251 cells, U251/TR cells, or hypoxic U251/TR cells, followed by TMZ treatment. **B**, **C** Number of colonies of U251 cells exposed to PBS or exosomes derived from U251 cells, U251/TR cells, or hypoxic U251/TR cells. **D**–**F** Expression of PKM2 in U251 cells with above treatment. **G** Viability of U251 cells treated with PBS or exosomes isolated from hypoxic shNC- or shPKM2-transfected U251/TR cells. Ns: no significance; **p < 0.01; and ****p < 0.0001

In this study, we explored the impact of exosomal PKM2 released by TMZ-resistant glioma cells on the sensitivity of TMZ in susceptible glioma cells. Exosomes derived from U251/TR cells markedly heightened the resistance of U251 cells to TMZ (Fig. 5A–C), which was further enhanced by exosomes from hypoxic U251/TR cells. However, treatment with U251 cell-derived exosomes had no effect on U251 cell growth. Furthermore, only exosomes derived from U251/TR cells and hypoxic U251/TR cells could increase PKM2 protein levels, but not mRNA (Fig. 5D–F). Exosomes derived from U251 cells did not affect PKM2 expression. We also examined whether exosomal PKM2 induced TMZ resistance in sensitive glioma cells. The activity of TMZ in U251 cells was evaluated, and exosomes were collected from hypoxic shNC- or shPKM2-transfected U251/TR cells (Additional file 1: Figure S2). Our data demonstrated that U251 cells treated with exosomes from hypoxic shPKM2-transfected U251/TR cells showed improved response to TMZ (Fig. 5G). Taken together, exosomes released from hypoxic TMZ-resistant glioma cells transfer PKM2 and contribute to acquired TMZ resistance in sensitive glioma cells.

### Hypoxic resistant glioma cells mitigate TMZ-induced apoptosis via delivering exosomal PKM2

**Fig. 6:**
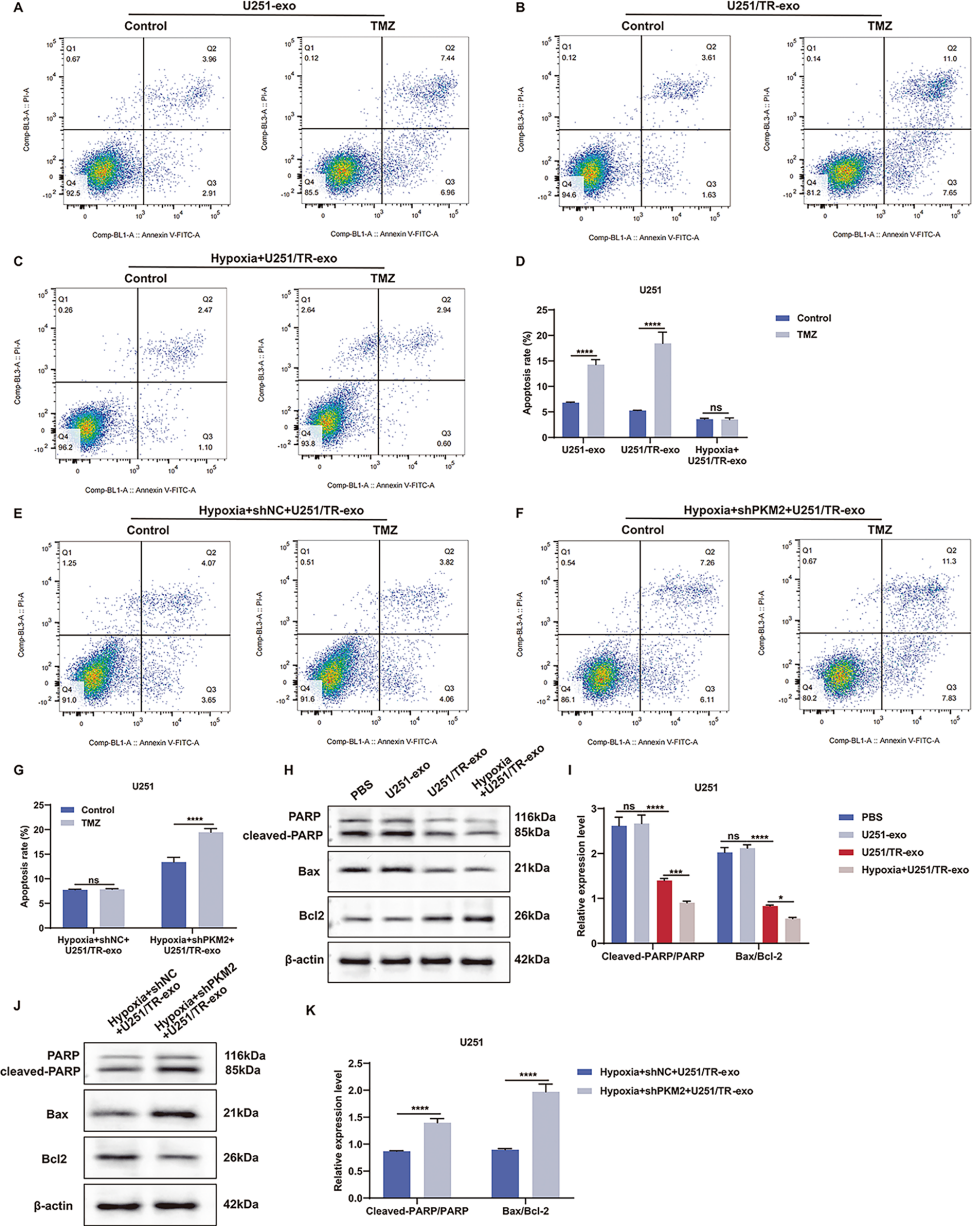
Hypoxic resistant glioma cells mitigate TMZ-induced apoptosis via delivering exosomal PKM2. **A**–**D** Apoptotic levels of U251 cells administrated with exosomes isolated from U251 cells, U251/TR cells, or hypoxic U251/TR cells, followed by TMZ. **E**–**G** Apoptotic levels of U251 cells treated with hypoxic shNC- or shPKM2-transfected U251/TR cells. **H**, **I** Cleaved PARP/PARP and Bax/Bcl-2 levels in U251 cells exposed to PBS, or exosomes from U251 cells, U251/TR cells, or hypoxic U251/TR cells. **J**, **K** Cleaved PARP/PARP and Bax/Bcl-2 levels in U251 cells administrated with exosomes derived from hypoxic shNC- or shPKM2-transfected U251/TR cells. Ns: no significance; *p < 0.05; ***p < 0.001; and ****p < 0.0001

In this study, we delved into the mechanisms through which exosomes originating from hypoxic TMZ-resistant glioma cells foster acquired TMZ resistance in glioma cells. We evaluated the extent of apoptosis in U251 cells when exposed to exosomes released from U251, U251/TR, or hypoxic U251/TR cells. The results demonstrated a significant increase in apoptosis in U251 cells when treated with TMZ in combination with exosomes derived from U251 or U251/TR cells (Fig. 6A–D). However, hypoxic U251/TR cell-released exosome administration did not change apoptosis levels in U251 cells under TMZ treatment. Moreover, exosomes from hypoxic shPKM2-transfected U251/TR cells were able to restore the capacity for facilitating apoptosis of U251 cells when exposed to TMZ (Fig. 6E–G). Cleaved PARP/PARP and Bax/Bcl-2 levels in U251 cells were not altered by U251 cell-derived exosomes (Fig. 6H, I). In contrast, the levels of these proteins decreased as a result of exosomes released from U251/TR cells, and this reduction was further pronounced by exosomes from hypoxic U251/TR cells. Furthermore, the effects mentioned above were reversed upon PKM2 suppression (Fig. 6J, K). Overall, exosomal PKM2 released by hypoxic resistant glioma cells reduces TMZ-induced apoptosis.

### Hypoxic resistant cell-derived exosomal PKM2 motivates intracellular ROS accumulation of sensitive glioma cells

**Fig. 7:**
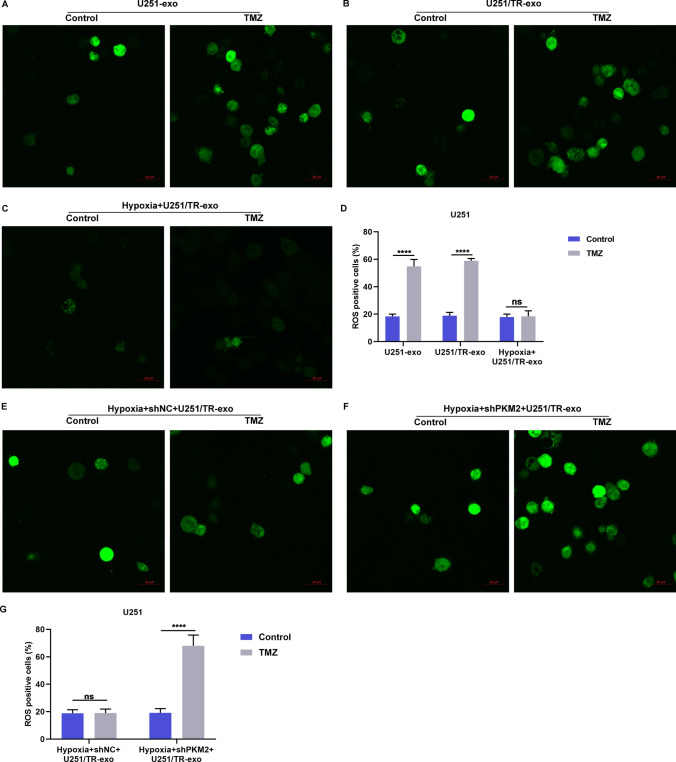
Hypoxic resistant cell-derived exosomal PKM2 motivates intracellular ROS accumulation of sensitive glioma cells. **A**–**D** ROS positive U251 cells treated with exosomes derived from U251 cells, U251/TR cells, or hypoxic U251/TR cells, followed by TMZ. Bar, 20 μm. **E**–**G** ROS positive U251 cells administrated with exosomes from hypoxic shNC- or shPKM2-transfected U251/TR cells. Bar, 20 μm. Ns: no significance; ****p < 0.0001

Previous studies have shown that reactive oxygen species (ROS) play a role in the resistance of glioma cells to TMZ [19]. In our study, we aimed to investigate whether exosomal PKM2 released from hypoxic resistant glioma cells affects intracellular ROS accumulation in sensitive glioma cells following TMZ treatment. We found that TMZ treatment led to a significant increase in intracellular ROS levels in U251 cells treated with exosomes derived from U251 or U251/TR cells, but not in those treated with exosomes from hypoxic U251/TR cells (Fig. 7A–D). Furthermore, in the presence of TMZ treatment, exosomes derived from hypoxic U251/TR cells demonstrated a reduction in intracellular ROS levels in U251 cells compared to those from U251 and U251/TR cells. However, the administration of exosomes from hypoxic U251/TR cells, which were transfected with PKM2, intensified ROS accumulation following TMZ treatment (Fig. 7E–G). Our findings indicate that delivery of exosomal PKM2 from hypoxic resistant glioma cells reduces intracellular ROS generation induced by TMZ in sensitive glioma cells.

### Hypoxic resistant cells transmit exosomal PKM2 to tumor-associated macrophages (TAMs) to facilitate TMZ resistance of glioma

**Fig. 8:**
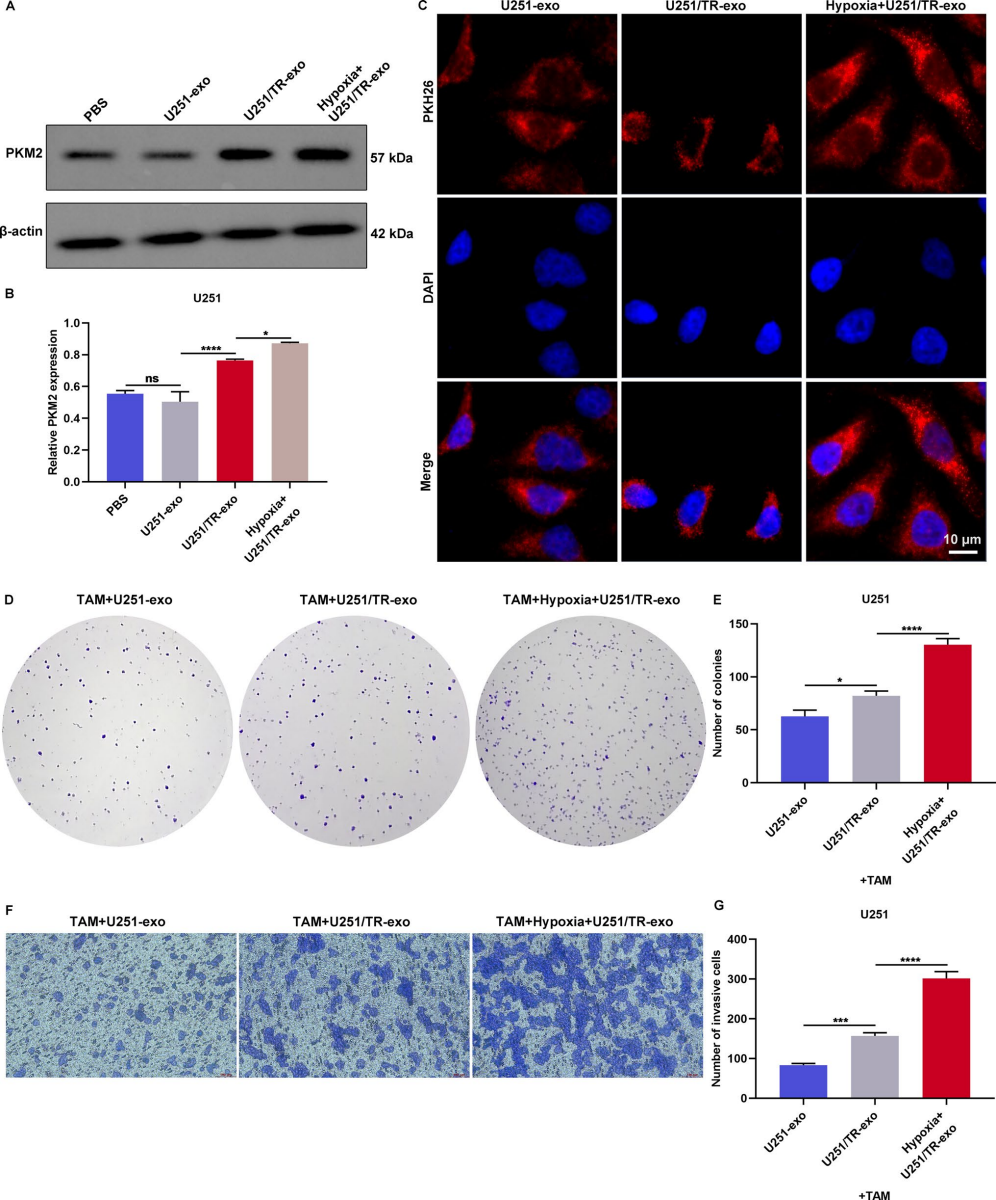
Hypoxic resistant cells transmit exosomal PKM2 to TAMs to facilitate TMZ resistance of glioma. **A**, **B** PKM2 expression in TAMs co-cultured with PBS, exosomes from U251 cells, U251/TR cells, or hypoxic U251/TR cells. **C** Red fluorescent dye PKH26 staining in TAMs co-cultured with exosomes from U251 cells, U251/TR cells, and hypoxic U251/TR cells for assessing exosome uptake. Bar, 10 μm. **D**, **E** Number of colonies of U251 cells cocultured with TAMs that were pretreated with exosomes from U251 cells, U251/TR cells, and hypoxic U251/TR cells after TMZ administration. **F**, **G** Number of invasive cells under above treatment. Bar, 100 μm. Ns: no significance; *p < 0.05; ***p < 0.001; and ****p < 0.0001

In this study, we examined the effects of different glioma cell-derived exosomes on TAMs. TAMs were co-cultured with exosomes obtained from U251 cells, U251/TR cells, or hypoxic U251/TR cells. Results showed that compared to exosomes derived from U251 cells, higher levels of PKM2 were observed in those from U251/TR cells, and this effect was further enhanced by hypoxic U251/TR cell-exosome treatment (Fig. 8A, B). Moreover, we found that TAMs could uptake exosomes released from U251 cells, U251/TR cells, or hypoxic U251/TR cells (Fig. 8C). TAMs that received exosomes from hypoxic U251/TR cells significantly increased the proliferative and invasive abilities of U251 cells following TMZ administration (Fig. 8D–G). These findings suggest that hypoxic resistant cells can transmit exosomal PKM2 to TAMs, consequently promoting TMZ resistance in glioma.

## Discussion

As the only FDA-approved oral agent for glioma therapy, TMZ plays a critical role as a DNA-alkylating agent which causes DNA double-strand breaks, cellular cycle arrest and ultimately cell death in glioma cells [20]. Despite its widespread use, TMZ remains limited in efficacy due to various factors such as extensive drug resistance, short blood-plasma half-life, rapid hydrolysis and imprecise tumor accumulation. Furthermore, over 50% of patients do not respond to TMZ, indicating that TMZ resistance remains a major challenge in glioma treatment [21]. Hypoxic condition deprives tissues of adequate oxygen supply, which is a main microenvironmental feature of glioma [22]. Hypoxia is a typical microenvironmental feature of glioma tissues characterized by insufficient oxygen supply. Hypoxic conditions are typically caused by rapid tumor cell proliferation, depleting available cellular nutrients and oxygen due to an inadequate blood supply. Hypoxic glioma cells show higher levels of aggressiveness, resistance to TMZ, and poorer clinical outcomes among glioma patients [23, 24]. As a result, hypoxia represents a major barrier that significantly reduces the therapeutic effect of TMZ in glioma treatment [25]. Our experimental evidence suggests that the hypoxic microenvironment exacerbates TMZ resistance in glioma cells. Moreover, PKM2 is involved in this process.

siRNA-based gene silencing has emerged as a powerful therapeutic strategy in recent years [26, 27]. Synthetic nanoparticles are currently the most commonly utilized siRNA delivery system, but their use is limited by several concerns such as toxicity, clearance issues, and complex synthesis methods [28]. Therefore, researchers have turned to endogenous nanomaterials like exosomes to enhance the efficacy and safety of siRNA delivery for glioma therapy. Exosomes offer unique advantages including autologous nature, immunosurveillance friendliness, multifunctional cargo delivery, and efficient BBB traversal capability [29]. In this study, we have uncovered the release of exosomal PKM2 from glioma cells exhibiting resistance to TMZ under hypoxic conditions. Our findings further indicate that the transmission of exosomal PKM2 from resistant glioma cells to sensitive counterparts can result in the acquisition of TMZ resistance and a reduction in apoptosis induced by this drug. Moreover, intracellular ROS accumulation in sensitive glioma cells is motivated by exosomal PKM2 released from resistant glioma cells, according to our results. As previously reported in literature, PKM2 has been demonstrated to induce the release of tumor cell-derived exosomes [30]. In colorectal carcinoma, exosome-mediated transmission of hsa_circ_0005963 has been found to enhance the glycolytic process and induce chemoresistance through post-transcriptional modulation of PKM2 axis [31]. Similarly, non-small cell lung carcinoma (NSCLC) cells that are resistant to cisplatin have been shown to transmit their resistance via exosomal PKM2 to sensitive cells [32]. NSCLC progression has also been revealed to accelerate due to exosomal circSHKBP1-induced glycolytic process involving PKM2 [33]. The metabolic switch of liver nonparenchymal cells has been shown to be mediated by exosomal PKM2 in activated hepatic stellate cells [34]. Additionally, it was demonstrated that exosomal PKM2 transmitted in plasma reinforced growth and motility in esophageal squamous cell carcinoma cells [35]. In prostate carcinoma, bone metastases were found to be motivated by exosomal PKM2 transmission into bone stroma [36]. Furthermore, exosomes have been shown to mediate the metabolic reprogramming of cancer-associated fibroblasts (CAFs), thereby contributing to improvement of the tumor microenvironment [37]. Lastly, gastric carcinoma cells have been shown to increase NF-κB signaling activation in CAFs through exosomal PKM2 secretion. This consequently motivated dysregulated metabolism and inflammation activation [38].

As a medical expert, it is crucial to acknowledge the pivotal role played by immune cells in the progression of glioma tumors. TAMs, as the most abundant immune cells, tend to accumulate preferentially in hypoxic areas and possess the potential to interact with tumor cells, leading to reprogramming [17]. In hypoxic glioma models, released exosomes are seen to induce the polarization of M2-like macrophages [22]. Additionally, studies suggest that exosomes containing Arginase-1 derived from reprogrammed TAMs may also facilitate glioma tumor progression [39]. Our current evidence indicates that exosomal PKM2 transmitted from hypoxic-resistant glioma cells can impact TAMs and enhance drug resistance to TMZ. Therefore, targeting PKM2 may offer a novel approach for treating TMZ-resistant glioma, laying the groundwork for future clinical applications. Collectively, these findings underscore the importance of comprehending the intercellular signaling mechanisms between tumors and immune cells in the development of effective treatment strategies for resistant glioma.

This study has several study limitations. One potential limitation of this study lies in the specificity of the glioma cell lines used to investigate the role of exosomal PKM2in TMZ resistance. Gliomas are a heterogeneous group of tumors with diverse genetic backgrounds, and the findings of this study may not fully capture the complexity of TMZ resistance in all glioma subtypes. Future research should consider employing a broader range of glioma cell lines and patient-derived models to enhance the generalizability of the results. Another limitation is the predominantly in vitro nature of the experimental design. While in vitro studies provide valuable insights into cellular mechanisms, the artificial conditions may not fully replicate the intricate tumor microenvironment present in vivo. Future investigations incorporating in vivo models or clinical data from glioma patients would be beneficial to validate the relevance of exosomal PKM2-mediated TMZ resistance in a more physiologically representative setting.

## Conclusion

Our study presents a new model in glioma that demonstrates how exosomes confer TMZ resistance. Through hypoxic microenvironment-induced exosomes, TMZ resistance is delivered through a PKM2-dependent mechanism. This crosstalk with TAMs resulted in the formation of a TMZ-resistant microenvironment. Consequently, exosomal PKM2 could be an effective marker for identifying clinically TMZ-resistant glioma and enabling targeted anti-PKM2 treatment for patients.

### Supplementary Information

**Additional file1: Figure S1.** Transmission electron microscopy was utilized to detect the separation of exosomes in various groups. **Figure S2.** Transmission electron microscopy was utilized to detect the separation of exosomes in various groups.

## Data Availability

The datasets analyzed during the current study are available from the corresponding author on reasonable request.

## Abbreviations

TMZ: Temozolomide
PKM2: Pyruvate kinase M2
BBB: Blood–brain barrier
FBS: Fetal bovine serum
CCK-8: Cell Counting Kit-8
OE-PKM2: Overexpressing PKM2
ROS: Reactive oxygen species
TAMs: Tumor-associated macrophages
