# Supplementary material for: Hypoxic microenvironment-induced exosomes confer temozolomide resistance in glioma through transfer of pyruvate kinase M2
Source: Discov Oncol. 2024 Apr 10;15:110. doi: 10.1007/s12672-024-00963-9 (PMC11006647; doi:10.1007/s12672-024-00963-9)
Supplement: Supplementary file 1 — Additional file1: Figure S1. Transmission electron microscopy was utilized to detect the separation of exosomes in various groups. Figure S2. Transmission electron microscopy was utilized to detect the separation of exosomes in various groups. [file 12672_2024_963_MOESM1_ESM.docx]

**Additional materials 1**


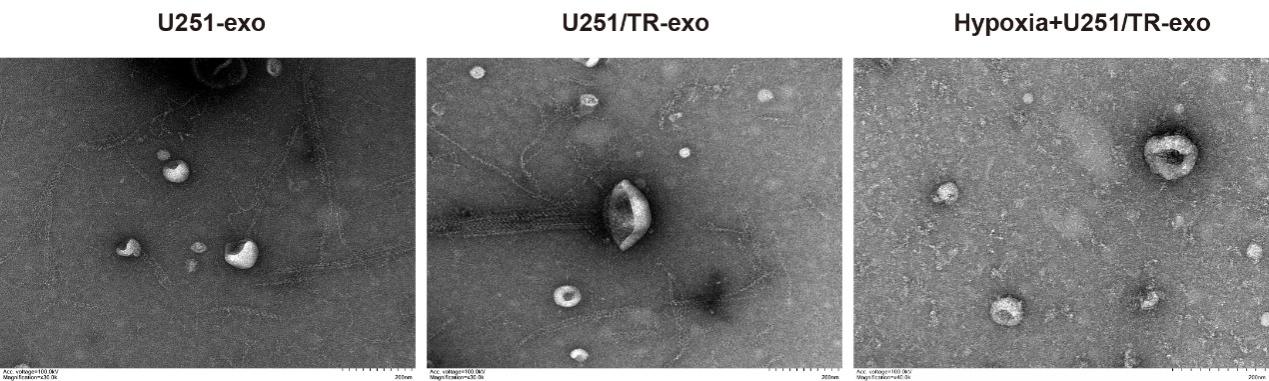


Additional Figure S1. Transmission electron microscopy was utilized to detect the separation of exosomes in various groups.


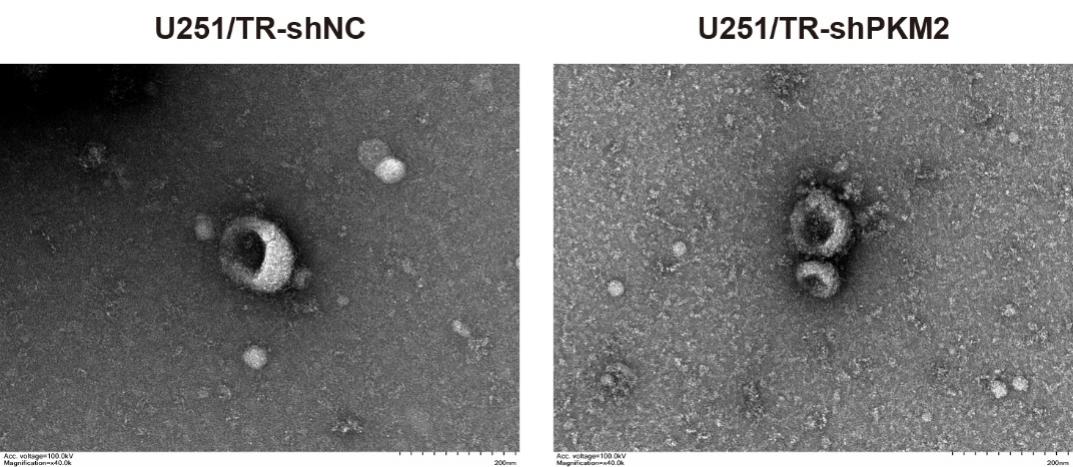


Additional Figure S2. Transmission electron microscopy was utilized to detect the separation of exosomes in various groups.
